# Gamma-Tocotrienol Modulated Gene Expression in Senescent Human Diploid Fibroblasts as Revealed by Microarray Analysis

**DOI:** 10.1155/2013/454328

**Published:** 2013-03-24

**Authors:** Suzana Makpol, Azalina Zainuddin, Kien Hui Chua, Yasmin Anum Mohd Yusof, Wan Zurinah Wan Ngah

**Affiliations:** ^1^Department of Biochemistry, Faculty of Medicine, Universiti Kebangsaan Malaysia, Jalan Raja Muda Abdul Aziz, 50300 Kuala Lumpur, Malaysia; ^2^Department of Physiology, Faculty of Medicine, Universiti Kebangsaan Malaysia, Jalan Raja Muda Abdul Aziz, 50300 Kuala Lumpur, Malaysia

## Abstract

The effect of **γ**-tocotrienol, a vitamin E isomer, in modulating gene expression in cellular aging of human diploid fibroblasts was studied. Senescent cells at passage 30 were incubated with 70 **μ**M of **γ**-tocotrienol for 24 h. Gene expression patterns were evaluated using Sentrix HumanRef-8 Expression BeadChip from Illumina, analysed using GeneSpring GX10 software, and validated using quantitative RT-PCR. A total of 100 genes were differentially expressed (*P* < 0.001) by at least 1.5 fold in response to **γ**-tocotrienol treatment. Amongst the genes were *IRAK3, SelS, HSPA5, HERPUD1, DNAJB9, SEPR1, C18orf55, ARF4, RINT1, NXT1, CADPS2, COG6*, and *GLRX5*. Significant gene list was further analysed by Gene Set Enrichment Analysis (GSEA), and the Normalized Enrichment Score (NES) showed that biological processes such as inflammation, protein transport, apoptosis, and cell redox homeostasis were modulated in senescent fibroblasts treated with **γ**-tocotrienol. These findings revealed that **γ**-tocotrienol may prevent cellular aging of human diploid fibroblasts by modulating gene expression.

## 1. Introduction

Aging is a phenomenon associated with gradual decline in biological functions. Loss of capability to divide besides loss of cellular functions in both mitotic and postmitotic cells is the characteristic of cellular aging [1]. Cellular changes that occur in the cell of an organism have direct impact on the functions of organs, systems and eventually involve the whole organism. The genetic theory of aging is proposed based on the observation that several genes affect longevity [2]. The aging process is regulated by specific genes in many organisms including yeast, *C. elegans*, fruit flies, and mice. In human diploid fibroblasts, several genes including inflammatory genes, cell cycle regulatory genes, cytoskeletal genes, and metabolic genes were differentially expressed [3] during replicative senescence and modifiable by dietary components such as antioxidants [4].

Human aging can be studied *in vitro*, specifically by using normal human diploid fibroblasts (HDFs) which undergo a limited number of cellular divisions in culture and progressively reached a state of irreversible growth arrest, a process termed as replicative senescence [1]. Senescent fibroblast cells are resistant to mitogen-induced proliferation, expressed senescence-associated *β*-galactosidase (SA *β*-gal), exhibited enlarged and flattened morphology, and showed altered gene expression [5]. Cultured human fibroblasts cells displayed age-dependent transcriptomic differences. A variety of genes involved in cell cycle regulation, immune response and inflammation, cytoskeleton, stress response, and metabolism are known to be altered during cellular senescence [6].

About 40 micronutrients consist of vitamins, essential minerals, and other compounds are required in small amount for normal metabolism and have been reported as essential components in the diet. Deficiency of macro- and micronutrients in aging is related to global impairments of immune functions, metabolic harmony, and antioxidant defense with subsequent appearance of age-related diseases [7]. The antioxidant vitamin E, usually alpha-tocopherol, scavenges reactive oxygen species (ROS) thus preventing oxidative damage associated with many degenerative diseases and has been suggested to act as a signaling molecule which modulates signal transduction and gene expression [8].

Vitamin E is a lipophilic vitamin, synthesized by plants, found particularly in plant seeds and oils. There are eight naturally occurring forms of vitamin E which are *α*-, *β*-, *γ*-, and *δ*-tocopherol and *α*-, *β*-, *γ*-, and *δ*-tocotrienol [9]. Structurally, tocotrienols are different from tocopherols by the presence of three trans double bonds in their hydrocarbon tail [10]. 

The prenyl side chain of tocotrienols has been postulated to be responsible for the differential membrane distribution and metabolism of tocotrienols. *α*-tocotrienol possessed higher antioxidant activity against lipid peroxidation than *α*-tocopherol due to a more uniform distribution in the lipid bilayer membrane providing a more efficient interaction of the chromanol ring with lipid radicals [11]. Tocotrienol also showed novel hypocholesterolemic activity [12] in addition to more recent reports suggesting that it has neuroprotective, antioxidant, anticancer and cholesterol lowering properties that often differ from the properties of tocopherols [10]. Furthermore, tocotrienol was able to delay cellular aging by preventing oxidative damage-induced telomere shortening in aged human fibroblast cells [13]. Also tocotrienol-rich fraction (TRF) has been shown to have antiaging properties by promoting cell cycle progression in senescent human fibroblast cells [5]. Recent research has focused on other biological functions of vitamin E that are unrelated to its antioxidant properties which include its roles in cellular signaling, gene expression, immune response, and apoptosis [9]. Therefore, this study was aimed to determine the effect of *γ*-tocotrienol in modulating gene expression in cellular aging of human diploid fibroblast cells.

## 2. Materials and Methods

### 2.1. Primary Culture of Human Diploid Fibroblast Cells and Treatment with *γ*-Tocotrienol

This research has been approved by the Universiti Kebangsaan Malaysia Ethics Committee (Approval Project Code: FF-104-2007). Informed written consent was obtained from the parents of all subjects. Primary human dermal fibroblasts were derived from the foreskins (removed during circumcision) of three 9-to 12-year-old boys. The samples were aseptically collected and washed several times with 75% alcohol and phosphate buffered saline (PBS) containing 1% antibiotic-antimycotic (PAA, Austria). After removing the epidermis, the pure dermis was cut into small pieces and transferred into falcon tubes containing 0.03% collagenase type I solution (Worthington Biochemical Corporation, USA). Pure dermis was digested in an incubator shaker at 37°C for 6–12 h. Then, cells were rinsed with PBS before being cultured in Dulbecco Modified Eagle Medium (DMEM) containing 10% foetal bovine serum (FBS) (PAA, Austria) and 1% antibiotic-antimycotic at 37°C in 5% CO_2_ humidified incubator. After 5-6 days, the cultured HDFs were harvested by trypsinization and culture-expanded in new T25 culture flasks (Nunc, Denmark) with expansion degree of 1 : 4. When the subcultures reached 80%–90% confluency, serial passaging was done by trypsinization, and the number of population doublings (PDs) was monitored until HDFs reached senescence. For subsequent experiments, cells were used at either passage 4 (young cells, PD < 12) and passage 30 (senescent cells, PD > 55). 

In the subsequent experiments, treated young HDFs were incubated with 50 *μ*M palm *γ*-tocotrienol (Malaysian Palm Oil Board), while senescent HDFs were incubated with 70 *μ*M *γ*-tocotrienol for 24 h. Untreated cells were cultured in Dulbecco Modified Eagle Medium (DMEM) containing 10% foetal bovine serum (FBS) (PAA, Austria). The media for the untreated cells were changed in parallel to the treated cells. Both untreated and treated cells were harvested on the same day [14].

### 2.2. Determination of Senescent Biomarker SA *β*-Gal Activity

The senescent biomarker of *in vitro* cell aging for HDFs (SA *β*-gal activity) was determined by senescent cells staining kit (Sigma, USA) according to the manufacturer's instruction. Blue staining was visible after 4 h of incubation with *β*-galactosidase staining solution containing 5-bromo-4-chloro-3-indolyl-*β*-D-galactosidase (X-gal) at 37°C. The percentage of blue cells observed in 100 cells under a light microscope was calculated.

### 2.3. Total RNA Extraction

Total RNA from HDFs in different treatment groups was extracted using TRI Reagent (Molecular Research Center, Cincinnati, OH) according to the manufacturer's instruction. Polyacryl carrier (Molecular Research Center) was added in each extraction to precipitate the total RNA. Extracted total RNA pellet was then washed with 75% ethanol and dried before being dissolved in RNase and DNase-free distilled water. Total RNA was stored at −80°C immediately after extraction. Concentration and purity of the extracted RNA were determined by Agilent 2100 bioanalyzer (Agilent Technologies, USA). RNA with RNA integrity number (RIN) ranging from 7 to 10 and absorbance ratio of A_260_ to A_280_ ranging from 1.5 to 2.1 was utilized for cDNA synthesis. 

### 2.4. Microarray Analysis

Briefly, 250 ng of total RNA from each sample was labeled by using TotalPrep RNA Amplification Kit (Ambion, Austin, USA) for cDNA synthesis. *In vitro *transcription was performed to synthesise the cRNA. Single stranded cRNA was labeled by incorporating biotin-16-UTP, and cRNA was generated by incubation in the hybridization oven for 14 h at 37°C. After 14 h incubation, 750 ng of biotin-labeled cRNA was hybridized (18 h at 58°C) to Illumina's Sentrix HumanRef-8_v3 Expression BeadChips (Illumina, San Diego, USA). The hybridized biotinylated cRNA was detected with streptavidin-Cy3 (Amersham Biosciences, USA) and quantitated using Illumina's BeadStation S455 scanner (Illumina, San Diego, USA). Data was analysed using GeneSpring GX 10 software (Agilent Technologies, USA), and two-way analysis of variance (2-way ANOVA) was applied with the false discovery rate (FDR) for the selection of differentially expressed genes at significance level of *P* < 0.001 for baseline (data not shown) and *γ*-tocotrienol-treated HDFs. Significant gene list was further filtered for differences of more than 1.5-fold between *γ*-tocotrienol-treated HDFs and untreated control senescent HDFs. Significant genes with expression greater than 1.5-fold were selected for Gene Set Enrichment Analysis (GSEA) by using pathway studio software (Ariadne, USA) with *P* < 0.05.

### 2.5. Validation of Microarray Data Using Quantitative Real-Time RT-PCR

Genes for validation were chosen from pathway analysis. Quantitative real-time RT-PCR reaction was carried out to evaluate the expression of *ARF4, HSPA5, *and *HERPUD1 *genes using 1 *μ*L total RNA as template, 1 *μ*L of forward and reverse primers for genes of interest, and iScript One-Step RT-PCR reagent with SYBR Green (Bio-Rad, USA). Primer sequences for *ARF4*, *HSPA5,* and *HERPUD1 *are shown in Table 1. All reactions were run in duplicate with reaction profile as follows: cDNA synthesis for 30 min at 50°C; predenaturation for 2 min at 94°C; PCR amplification for 38 cycles with 10 sec at 94°C and 30 sec at 61°C using Bio-Rad iCycler (Bio-Rad, USA).

## 3. Results

### 3.1. Quality Control Assessment of the Samples

**Figure 1:**
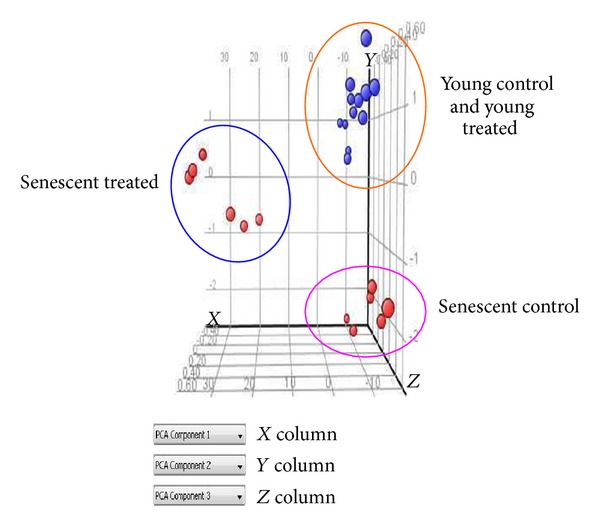
Three-dimensional Principal Component Analysis (PCA) plot was derived from biological replicates of HDFs (*n* = 6). The three dimensional PCA plot showed clustering of the different treatment groups: untreated control senescent HDFs, *γ*-tocotrienol-treated senescent HDFs, and untreated control young HDFs and *γ*-tocotrienol-treated young HDFs.

Principal Component Analysis (PCA) was used to check the quality of the microarray data. This allows viewing of separation between groups of replicates. Untreated control senescent and *γ*-tocotrienol-treated senescent HDFs were well separated and clustered into two distinct groups. Similar separation, however, was not observed for untreated control young and *γ*-tocotrienol-treated young HDFs (Figure 1).

### 3.2. Hierarchical Clustering of Significantly Expressed Genes

**Figure 2:**
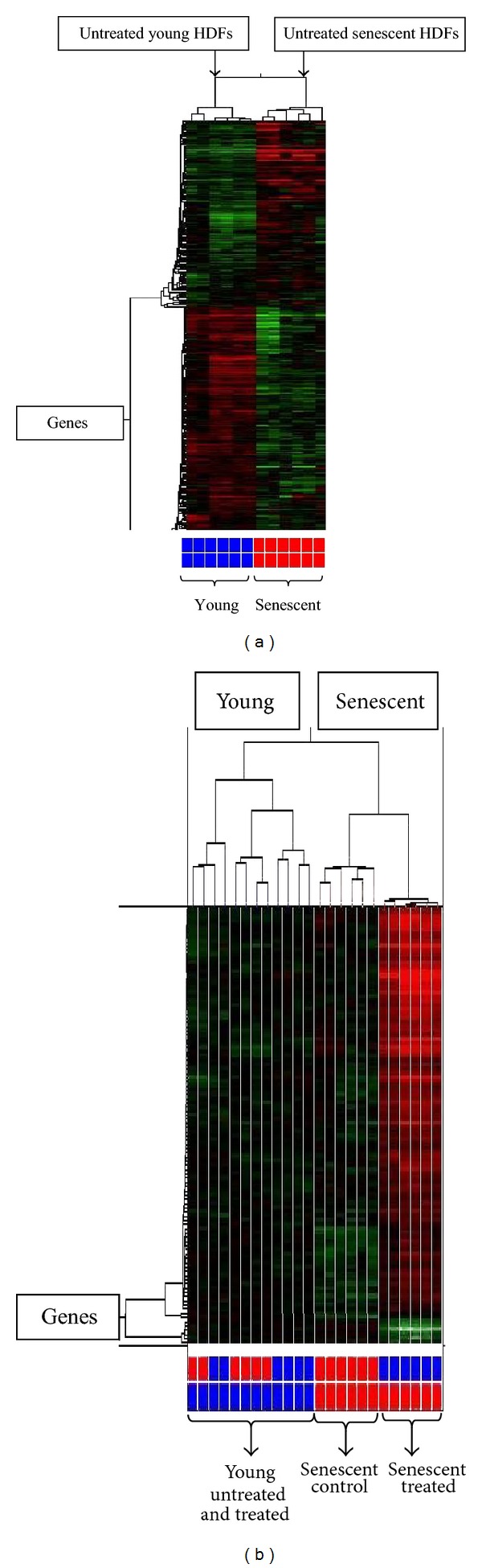
Hierarchical cluster analysis of genes showed the differential expression of genes in young and senescent HDFs (a) and senescent HDFs in response to *γ*-tocotrienol treatment (b) (*P* < 0.001). Samples were clustered under their conditions.

Cluster analysis was performed to organize genes into cluster based on their similarities of expression. Horizontal line represents a single gene, and each column represents a single sample. Red color indicated the upregulated genes, whereas green color indicated the downregulated genes. Statistical analysis of two-way analysis of variance (2-way ANOVA) revealed that a total of 253 genes were significantly regulated in senescent HDFs as compared to young cells (Figure 2(a)). One hundred genes were significantly regulated in tocotrienol-treated senescent HDFs compared to untreated control senescent HDFs (Figure 2(b)). Clustering analysis was able to distinguish gene expression between young and senescent HDFs as well as between untreated control senescent and tocotrienol-treated senescent HDFs as shown by Hierarchical clustering.

### 3.3. Analysis of Differentially Expressed Genes in *γ*-Tocotrienol-Treated HDFs

**Figure 3:**
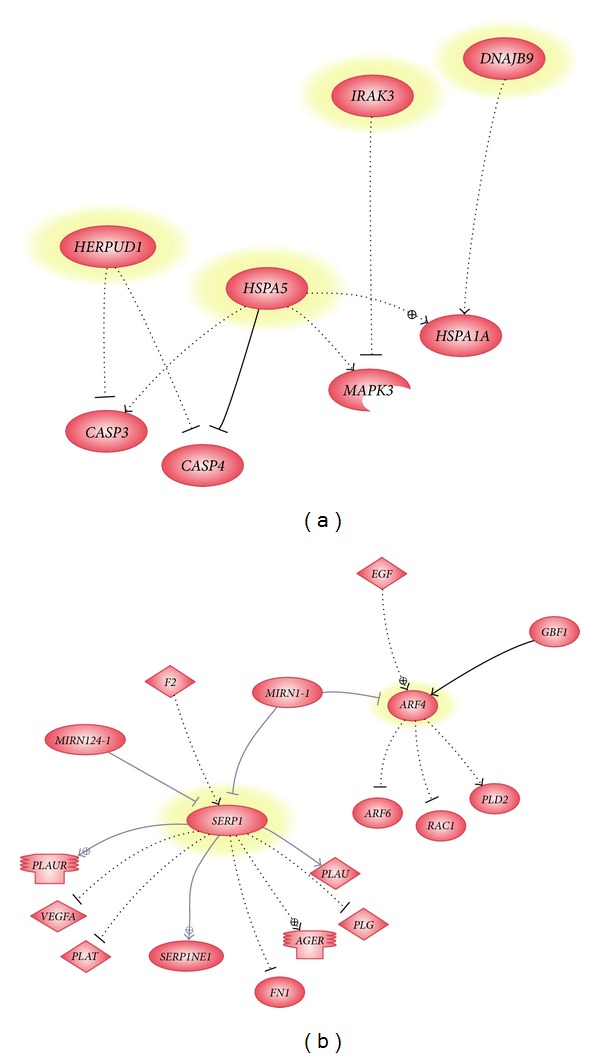
Network of common target for the molecular relationship of selected differentially expressed genes in *γ*-tocotrienol-treated senescent HDFs. The highlighted genes were the regulator for the downstream genes.

**Figure 4:**
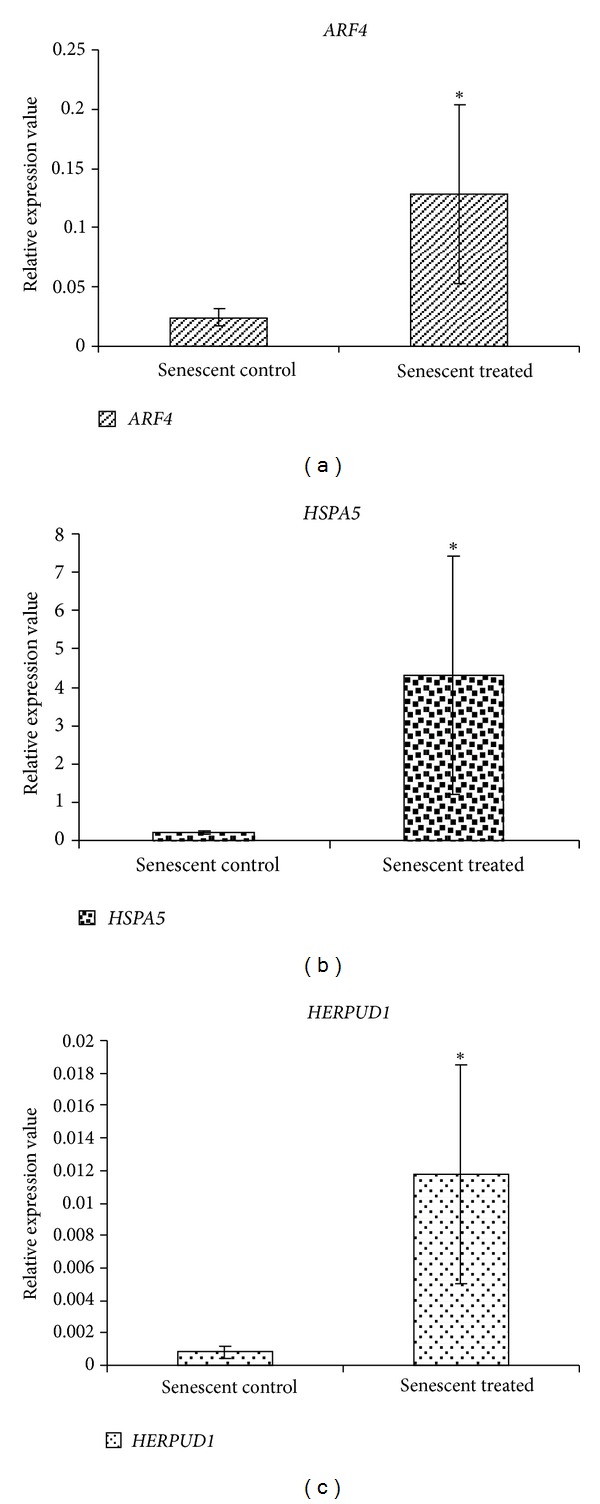
Based on pathway analysis, three genes were selected for validation by qRT-PCR technique. All data were normalized to the housekeeping gene, *GAPDH*. The expression patterns obtained through qRT-PCR were consistent with the microarray results.* ARF4* (a), *HSPA5* (b), and *HERPUD1* (c) were significantly upregulated in *γ*-tocotrienol-treated senescent HDFs compared to untreated control senescent HDFs (*P* < 0.05).

A total of 100 genes were differentially expressed in *γ*-tocotrienol-treated senescent HDFs as compared to untreated control senescent HDFs (Table 2). Gene Set Enrichment Analysis (GSEA) revealed the selected significant biological processes involved in response to *γ*-tocotrienol treatment in senescent HDFs compared to untreated control senescent HDFs (Table 3). Positive value of Normalized Enrichment Score (NES) indicated the upregulated process. The biological processes that were modulated by *γ*-tocotrienol treatment included the negative regulation of tumor necrosis factor production (inflammation), negative regulation of interleukin-6 production (inflammation), negative regulation of caspases activity (apoptosis), response to stress, transport protein, and cell redox homeostasis. Pathway analysis was carried out for selected genes (*ARF4, HSPA5,* and *HERPUD1*) (Figure 3), and validation on the microarray data was carried out by real time RT-PCR. Results showed an up-regulation of the selected genes in *γ*-tocotrienol-treated senescent HDFs as compared to untreated control senescent HDFs (Figure 4). Comparison between microarray and RT-PCR data showed that there was a consistent expression pattern of *ARF4, HSPA5,* and *HERPUD1* genes which are involved in protein transport and negative regulation of apoptosis (Table 4). 

## 4. Discussion

Nonantioxidant activities of vitamin E particularly *α*-tocopherol has been increasingly reported. For instance, *α*-tocopherol and *α*-tocopheryl phosphate were reported to be involved as mediators of lipid metabolism by modulating signal transduction and gene expression [8]. Our findings from the present study showed that another form of vitamin E, *γ*-tocotrienol, was able to modulate gene expression in human diploid fibroblasts. In response to *γ*-tocotrienol treatment, a total of 100 genes were differentially expressed in senescent HDFs which included *IRAK3, SelS, HSPA5, HERPUD1, DNAJB9, SEPR1, C18orf55, ARF4, RINT1, NXT1, CADPS2, COG6, *and* GLRX5.* Gene Set Enrichment Analysis (GSEA) revealed that *IRAK3* was involved in inflammation process specifically in the negative regulation of tumor necrosis factor production and negative regulation of interleukin-6 production. *IRAK3* encodes for one of the interleukin receptor-associated kinase (IRAK) family and has a role in both positive and negative regulation of signal transduction. This gene is also known as *IRAK-M,* and previously, the expression of human *IRAK-M* was limited to monocytes and macrophages [15], However in the present study, its expression was significantly increased in *γ*-tocotrienol-treated senescent fibroblast cells. 

The aging process is attributed to the presence of low chronic inflammation resulting in a stressed condition. Genes related to inflammation are relevant after taking into account that the innate immunity is more involved during inflammation. A chronic inflammatory status called inflame aging appears to be the major component of common age-related diseases including cardiovascular diseases and infections [16]. Among the inflammatory agents that have been identified were interleukin-6, interleukin-1*β*, cyclooxygenase 2 and tumor necrosis factor (TNF). Up-regulation of proinflammatory mediators was observed during aging due to an age-related redox imbalance that activates several proinflammatory signaling pathways. Dysregulation of these cytokines promotes inflammation and tissue damage. This indicated that aging is accompanied by chronic low-grade inflammation state showed by 2-to 4-fold increase in serum levels of inflammatory mediators such as C-reactive protein, tumor necrosis factor (TNF) and cyclooxygenase 2 (COX2) [17]. Up-regulation of *IRAK3* in this study may indicate protection against cellular aging and age-related diseases. 

In the present study we also found that selenoprotein* S* (*SelS*) was significantly upregulated in *γ*-tocotrienol-treated senescent fibroblast cells. Selenoprotein S is involved in negative regulation of tumor necrosis factor production, negative regulation of interleukin-6 production, and cell redox homeostasis. It encodes for an endoplasmic reticulum (ER) transmembrane protein and is present in a variety of tissues such as liver, skeletal muscle, and adipose tissue [18]. Selenoproteins contain the twenty-first amino acid, selenocysteine, reported to be involved in cellular defenses against oxidative damage. Furthermore, these proteins are involved in important metabolic and developmental pathways in response to environmental challenges [19]. Many of the selenoproteins are involved in protection against oxidative stress or in maintaining cellular redox balance. SelS is considered as an important component of retrotranslocation channel in endoplasmic reticulum-associated protein degradation (ERAD). This ER membrane protein functions in stress responses to prevent the deleterious consequences of accumulation of misfolded proteins which has been linked to immune and inflammatory processes. Previous findings have suggested that SelS may regulate cytokine production in macrophages, and a regulatory loop between cytokines and SelS has been proposed to play a key role in controlling the inflammatory response [20]. Negative regulation of TNF and interleukin-6 production by *IRAK3* and *SelS* in *γ*-tocotrienol-treated senescent HDFs observed in this study may indicate inhibition of chronic inflammatory processes that normally accompanies cellular aging. Thus this may suggest one of the mechanisms involved for *γ*-tocotrienol in slowing down cellular aging of HDFs.

*γ*-Tocotrienol also modulated the expression of genes that are involved in protein transport. Our results showed that ADP-ribosylation factor 4 (*ARF4*) was significantly upregulated in *γ*-tocotrienol-treated senescent HDFs. ADP-ribosylation factors have been implicated in several important cellular processes, including membrane trafficking and activation of phospholipase D [21]. They are members of the Ras super family of small guanine nucleotide-binding proteins and were initially identified as proteins that stimulate the ADP-ribosyl transferase activity of cholera toxin *in vitro*. ARFs are ubiquitously expressed from yeast to mammals and function primarily in the regulation of membrane trafficking. Based on their deduced amino acid sequence, protein size, and phylogenetic analysis, these proteins can be divided into three groups which are class I (ARF1, ARF2, and ARF3), class II (ARF4, ARF5), and class III (ARF6). Previous report has demonstrated that overexpression of ARF4 in U373MG cells suppresses N-(4-hydroxyphenyl) retinamide (4-HPR)-induced cell death. The findings from yeast-based functional screening in *S. cerevisiae* which facilitates the identification of antiapoptotic mammalian genes showed that ARF4 may function as a novel suppressor of apoptosis [22].

Heat shock proteins (Hsps) have been studied for many years, and there are evidences that demonstrated the role of Hsp up-regulation in tissues and cell protection in a wide variety of stress conditions. Heat shock proteins (HSPs) belong to a class of highly conserved proteins that act physiologically as molecular chaperones to stabilize existing proteins against aggregation and mediate the folding of newly translated proteins in the cytosol and other organelles [23]. Additionally, they have been shown to demonstrate antiapoptotic effects against a wide range of both physical and chemical apoptotic stimuli [24]. Similar finding was found in our study, whereby the *HSPA5* (heat shock 70 kDa protein 5) also known as BiP or GRP78 (glucose-regulated protein, 78 kDa) which is involved in negative regulation of caspase activity in response to stress was significantly increased in *γ*-tocotrienol-treated senescent HDFs. Besides, treatment with *γ*-tocotrienol in senescent HDFs caused up-regulation of *HERPUD1* which is also involved in negative regulation of caspase activity in response to stress. HERP (homocysteine-induced ER protein) which was recently identified and characterized as an ER membrane protein was upregulated in response to ER stress. Induction of HERP was reported to be involved in the protection of cells against ER stress. HERP stabilizes ER Ca^2+^ homeostasis and mitochondrial functions in neuronal cells during ER stress [25]. Thus the findings from the present study showed that treatment with *γ*-tocotrienol in senescent HDFs may delay cellular aging of HDFs by modulating cellular stress responses and regulating the apoptosis pathway.

Oxidative stress is known to be involved in a number of pathological conditions, including neurodegeneration, cardiovascular disease, and stroke, and even plays a role in natural aging. Oxidative stress occurs when the levels of oxidants are higher than the levels of antioxidants, thus overwhelming the system. The imbalance between prooxidants and antioxidants leads to an accumulation of oxidative damage with age in a variety of macromolecules resulting in a progressive loss of functional cellular processes, leading to the aging phenotype [26]. A progressive rise of oxidative stress due to the altered redox homeostasis appears to be one of the hallmarks of the aging process. Changes in the pattern of gene expression through ROS-sensitive transcription factors give rise to both aging and inflammation phenotypes. Chronic oxidative stress and inflammatory reactions lead to many age-associated diseases such as atherosclerosis and arthritis [27].

Cells have developed both nonenzymatic and enzymatic defense mechanisms to counteract the deleterious effects of oxidative stress by either detoxifying reactive oxygen species (ROS) or repairing ROS-induced damage. Nonenzymatic examples of antioxidant include vitamin C, vitamin E, ubiquinone, flavonoids and glutathione (GSH), and examples of enzymatic scavengers include catalase, glutathione peroxidase, thioredoxin, Cu/Zn superoxide dismutase (Cu/Zn SOD), Mn/superoxide dismutase (MnSOD), and glutaredoxins [28]. Interestingly, our finding showed that *γ*-tocotrienol intervention in senescent HDFs caused increased expression of glutaredoxin 5 homolog (*GLRX5*) gene which is involved in cell redox homeostasis. Glutaredoxins are glutathione-dependent oxidoreductases that help in maintaining cellular redox homeostasis in the cell. The glutaredoxin system consists of GSH, NADPH, and GSH reductase. The mammalian system contains three known members of the glutaredoxin family Grx1, Grx2, and Grx5. The recently discovered glutaredoxin 5, a monothiol glutaredoxin, is hypothetically localized to the mitochondria. The loss of Grx5 in yeast leads to constitutive oxidative damage, sensitization to ROS, iron accumulation and inactivation of iron-sulfur (Fe-S) cluster containing enzymes [29]. Our findings are in agreement with a recent study that showed overexpression of glutaredoxin 5 caused decreased in DNA fragmentation and protect cells against H_2_O_2_ induced apoptosis and ROS formation [30]. 

In summary, the findings from this study elucidated the nonantioxidant properties of a vitamin E isomer, *γ*-tocotrienol in delaying cellular aging indicated by regulation of protective biological processes through the modulation of gene expression in human diploid fibroblasts.

## 5. Conclusion

*γ*-Tocotrienol may delay or protect against cellular aging of human diploid fibroblasts by modulating genes expression that are involved in biological processes related to oxidative stress such as inflammation, protein transport, apoptosis, and cell redox homeostasis.

## Figures and Tables

**Table 1:** Primers list for qRT-PCR.

Gene	Accession number	Primer sequences (5′-3′)	PCR product size (bp)
*HSPA5 *	NM_005347	F: ggt gaa aga ccc ctg aca aa	199
		R: gtc agg cga ttc tgg tca tt	
*HERPUD1 *	NM_001010990	F: gag cct gct ggt tct aat cg	193
		R: gaa agc tga agc cac cca ta	
*ARF4 *	NM_001660	F: ggg atg ttg gtg gtc aag at	168
		R: agc agc act gca tct ctc aa	

**Table 2:** Differentially expressed genes list in *γ*-tocotrienol-treated senescent HDFs.

Gene Ontology/accession number	Gene symbol	Fold change	Regulation
Protein transport			
NM_020751.1	COG6	2.9372108	Upregulated
NM_022374.1	ARL6IP2	2.4375103	Upregulated
NM_017954.8	CADPS2	2.4749646	Upregulated
NM_004794.2	RAB33A	4.2599683	Upregulated
NM_001660.2	ARF4	3.6646543	Upregulated
NM_021930.3	RINT-1	1.6876434	Upregulated
NM_001031677.2	RAB24	2.3763354	Upregulated
NM_013248.2	NXT1	3.5031867	Upregulated
NM_014177.1	C18orf55	1.5897815	Upregulated
NM_017986.2	GPR172B	2.2754004	Upregulated
Ion transport			
NM_000725.2	CACNB3	2.0575228	Downregulated
NM_002245.2	KCNK1	4.019881	Upregulated
NM_022055.1	KCNK12	2.6714919	Upregulated
NM_012463.2	ATP6V0A2	2.7435005	Upregulated
NM_005175.2	ATP5G1	2.527163	Upregulated
NM_000785.3	CYP27B1	7.801915	Upregulated
Negative regulation of interleukin-6 production			
NM_203472.1	SELS	2.317949	Upregulated
NM_007199.1	IRAK3	2.630288	Upregulated
Immune response			
NM_002162.2	ICAM3	3.910169	Upregulated
Cytokine mediated signal transduction			
NM_203453.2	PPAPDC2	1.8218696	Downregulated
Apoptosis			
NM_018530.1	GSDML	2.4119947	Upregulated
NM_018130.2	SHQ1	1.8244729	Upregulated
NM_018456.4	EAF2	7.062471	Upregulated
NM_018145.1	FAM82C	1.6341798	Upregulated
NM_024310.2	PLEKHF1	2.9315765	Upregulated
NM_001012398.1	AKTIP	2.6247795	Upregulated
Response to stress			
NM_005347.2	HSPA5	5.951358	Upregulated
NM_021237.3	SELK	3.3043954	Upregulated
NM_014445.2	SERP1	3.263752	Upregulated
Cell redox homeostasis			
NM_016417.2	GLRX5	1.7182248	Upregulated
Cell cycle			
NM_006545.4	TUSC4	5.8936715	Upregulated
NM_016948.1	PARD6A	2.2347567	Upregulated
Regulation of transcription			
NM_005444.1	RQCD1	2.0665886	Upregulated
NM_004634.2	BRPF1	1.8511329	Upregulated
NM_003408.1	ZFP37	2.5321555	Upregulated
NM_015394.4	ZNF10	2.5174437	Upregulated
NM_001080485.1	ZNF275	1.8915362	Upregulated
NM_005088.2	CXYorf3	2.2247052	Upregulated
NM_032758.3	PHF5A	2.0002973	Upregulated
NM_022366.1	TFB2M	2.2978535	Upregulated
NM_145805.1	ISL2	2.0560906	Upregulated
Protein binding			
NM_021934.3	C12orf44	2.943061	Upregulated
NM_181291.1	WDR20	1.9003692	Upregulated
NM_006207.1	PDGFRL	3.129943	Upregulated
Protein folding			
NM_021800.2	DNAJC12	4.182389	Upregulated
Endoplasmic reticulum unfolded protein response			
NM_001010990.1	HERPUD1	6.5408597	Upregulated
Regulation of RhoGTPase activity			
NM_153213.2	ARHGEF19	2.899712	Upregulated
Regulation of rab GTPase activity			
NM_022771.3	TBC1D15	2.361947	Upregulated
NM_020705.1	TBC1D24	3.0267975	Upregulated
Cell-cell signalling			
NM_001005914.1	SEMA3B	1.764685	Upregulated
NM_032331.2	MGC2408	2.6944642	Upregulated
NM_001407.1	CELSR3	3.9751458	Upregulated
Translation			
NM_181463.1	MRPL55	3.1179419	Upregulated
NM_003136.2	SRP54	2.6215558	Upregulated
Electron transport chain			
NM_025147.2	COQ10B	1.7724766	Upregulated
NM_007022.3	CYB561D2	3.542795	Upregulated
Signal transduction			
NM_133173.2	APBB3	2.0743015	Upregulated
NM_016115.3	ASB3	2.5060174	Upregulated
Cell proliferation			
NM_206825.1	GNL3	4.2791877	Upregulated
DNA replication			
NM_017443.3	POLE3	3.112311	Upregulated
Oxidoreductase			
NM_017758.2	ALKBH5	3.526197	Upregulated
Microtubule sitoplamic organization			
NM_014171.3	CRIPT	2.496879	Upregulated
Growth			
NM_031479.3	INHBE	24.436758	Upregulated
Biosynthesis process			
NM_014305.1	TGDS	2.4665043	Upregulated
NM_133443.1	GPT2	6.0515475	Upregulated
NM_005768.5	OACT5	2.454743	Downregulated
Modification-dependent protein catabolic process			
NM_203301.1	FBXO33	1.5563589	Upregulated
NM_015984.1	UCHL5	2.2333875	Upregulated
Hydrolase activity			
NM_203453.2	PPAPDC2	1.8218696	Downregulated
Proteolysis			
NM_032549.1	IMMP2L	1.596754	Downregulated

**Table 3:** Selected significant biological processes that were modulated in senescent HDFs after *γ*-tocotrienol treatment for 24 h.

Biological process	Gene Set Enrichment Analysis (GSEA)		
	Normalized Enrichment Score	Gene symbol	Description
Negative regulation of tumor necrosis factor production	1.54164		
		IRAK3	Interleukin-1 receptor-associated kinase 3
		Sels	selenoprotein S

Negative regulation of interleukin-6 production	1.65684		
		IRAK3	Interleukin-1 receptor-associated kinase 3
		Sels	selenoprotein S

Negative regulation of caspase activity	1.40798		
		HSPA5	Heat shock 70 kDa protein 5 (glucose-regulated protein, 78 kDa)
		HERPUD1	Homocysteine-inducible, endoplasmic reticulum stress-inducible, ubiquitin-like domain member 1

Response to stress	1.61171		
		HSPA5	Heat shock 70 kDa protein 5 (glucose-regulated protein, 78 kDa)
		HERPUD1	Homocysteine-inducible, endoplasmic reticulum stress-inducible, ubiquitin-like domain member 1
		DNAJB9	DnaJ (Hsp40) homolog, subfamily B, member 9
		SERP1	Stress-associated endoplasmic reticulum protein 1

Protein transport	1.68316		
		C18orf55	Chromosome 18 open reading frame 55
		ARF4	ADP-ribosylation factor 4
		RINT1	RAD50 interactor 1
		SERP1	Stress-associated endoplasmic reticulum protein 1
		NXT1	NTF2-like export factor 1
		CADPS2	Ca^2+^—dependent activator protein for secretion 2
		COG6	Component of oligomeric golgi complex 6

Cell redox homeostasis	1.56752		
		GLRX5	Glutaredoxin 5 homolog
		Sels	Selenoprotein S

**Table 4:** Comparison between microarray and qRT-PCR data.

Gene	Fold change (RT PCR)	Fold change (microarray)	Regulation
ARF4	5.275	3.66465	Upregulated
HERPUD1	14.26	6.54086	Upregulated
HSPA5	20.99	5.95136	Upregulated
